# Prevention of Ischemic Myocardial Contracture Through Hemodynamically Controlled DCD

**DOI:** 10.1007/s13239-021-00537-8

**Published:** 2021-04-29

**Authors:** Ylva Wahlquist, Kristian Soltesz, Qiuming Liao, Xiaofei Liu, Henry Pigot, Trygve Sjöberg, Stig Steen

**Affiliations:** 1 Department of Automatic Control, Lund University, Lund, Sweden; 2 Division of Thoracic Surgery, Department of Clinical Sciences, Lund University, Lund, Sweden; 3 Department of Cardiothoracic Surgery, Skåne University Hospital, Lund, Sweden; 4 First Affiliated Hospital of Zhengzhou University, Zhengzhou, China

**Keywords:** DCD, Organ preservation, Ischemic damage, Hemodynamic control, Closed-loop drug administration

## Abstract

**Purpose:**

Ischemic myocardial contracture (IMC) or “stone heart” is a condition with rapid onset following circulatory death. It inhibits transplantability of hearts donated upon circulatory death (DCD). We investigate the effectiveness of hemodynamic normalization upon withdrawal of life-sustaining therapy (WLST) in a large-animal controlled DCD model, with the hypothesis that reduction in cardiac work delays the onset of IMC.

**Methods:**

A large-animal study was conducted comprising of a control group ($n=6$) receiving no therapy upon WLST, and a test group ($n=6$) subjected to a protocol for fully automated computer-controlled hemodynamic drug administration. Onset of IMC within 1 h following circulatory death defined the primary end-point. Cardiac work estimates based on pressure-volume loop concepts were developed and used to provide insight into the effectiveness of the proposed computer-controlled therapy.

**Results:**

No test group individual developed IMC within ${1} \text { h}$, whereas all control group individuals did (4/6 within ${30}{\text { min}}$).

**Conclusion:**

Automatic dosing of hemodynamic drugs in the controlled DCD context has the potential to prevent onset of IMC up to ${1}{\text { h}}$, enabling ethical and medically safe organ procurement. This has the potential to increase the use of DCD heart transplantation, which has been widely recognized as a means of meeting the growing demand for donor hearts.

**Supplementary Information:**

The online version contains supplementary material available at 10.1007/s13239-021-00537-8.

## Introduction

### Ischemic Myocardial Contracture

**Figure 1:**
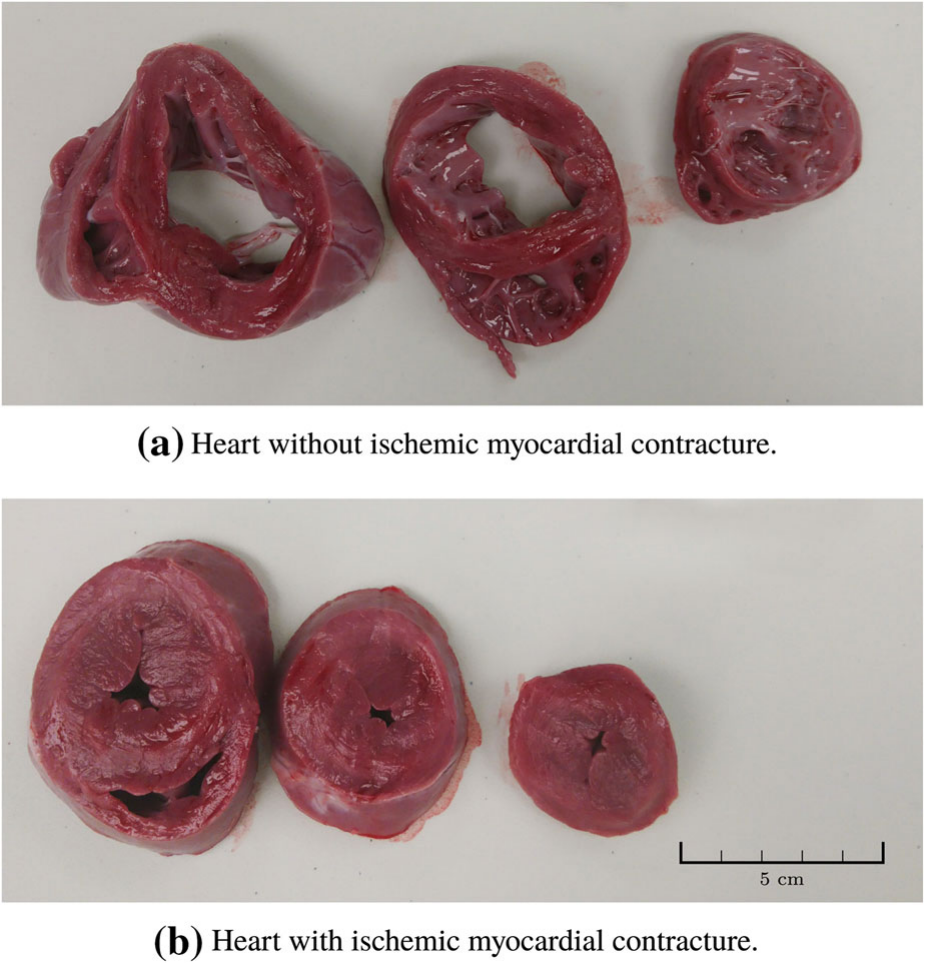
Transverse sections of two hearts from 35 kg pigs. Heart (a) was procured ${1}{\text { h}}$ after circulatory death, from one of the test group animals; (b) was procured ${30}{\text { min}}$ after circulatory death, from one of the control group animals. Notice that the left-ventricular lumen is almost gone in the contracted heart. Both photos are in the same scale, indicated in (b).

Ischemic myocardial contracture (IMC), commonly referred to as *stone heart*, develops when the myocardium is exerting mechanical work under warm ischemic conditions.^1^,^4^ The contracture commences at the apex of the heart, and subsequently extends throughout the left heart, before also affecting the right heart. Figure 1 shows cross sections of (a) one heart without, and (b) one with IMC. The contracture prevents the affected myocardium from performing mechanical work.

During the early era of open-heart surgery using cardiopulmonary bypass, IMC was identified as a rare, but fatal condition.1 Since it is associated with a loss of perfusion of the affected myocardium, the condition can typically not be reversed, as it prevents transport of required pharmacological substances to the affected site. Several works^21^,^3^ have investigated preventive measures. Administration of $\beta$-blockers, calcium antagonists and regional hypothermia, have all been shown to significantly reduce the risk of IMC.^1^,^4^

The advent of modern cardioplegia and general methodology development within cardiopulmonary bypass surgery have resulted in fewer instances of IMC. Consequently, the research interest in prevention of ischemic myocardial contracture has also decreased over the last three decades.

### Controlled Donation Upon Circulatory Death (DCD)

The inability to meet the demand of transplantable solid organs through donation upon brain death (DBD) from heart-beating brain-dead donors, has led to the reintroduction of donation upon circulatory death (DCD) in several legislations.^8^,^10^ Ischemic damage, resulting in graft failure, is the main medical concern associated with DCD transplantation.^9^,^2^ Procurement of DCD hearts is therefore performed under tight temporal constraints, and with maximal effort spent to prevent ischemic myocardial damage.^2^,^7^ This has limited its clinical application to Maastricht category III donors.^6^ This category constitutes in-hospital patients, where a decision to end life-sustaining ventilator support is based on the best interest of the patient.

As opposed to the determination of brain death, there exist no universally accepted criteria for the determination of circulatory death. Instead, its definition relies on the concepts of cessation and irreversibility of cardiopulmonary function.^19^ This has resulted in substantial DCD protocol variations between centra.^8^

**Figure 2:**
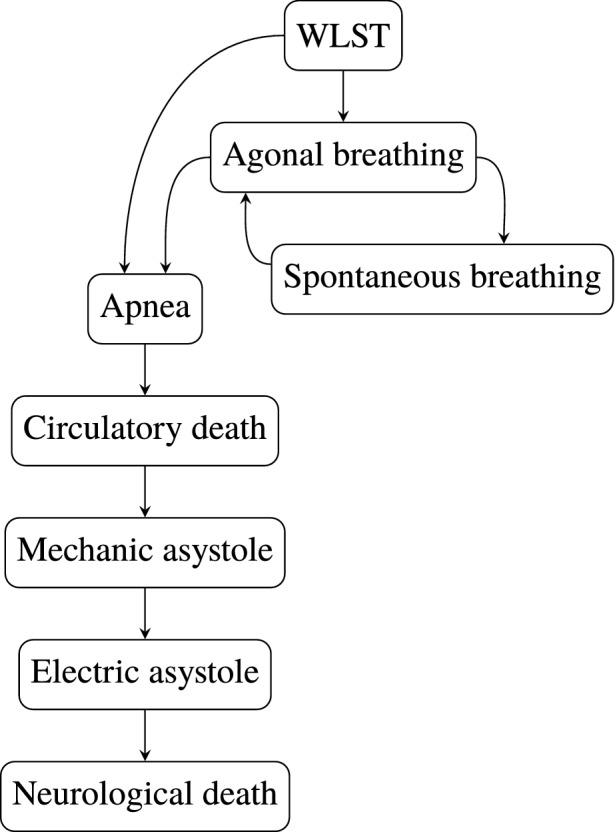
The course of events following withdrawal of life-sustaining therapy (WLST). Contemporary protocols prevent DCD donation where prolonged episodes (hours to days) are spent in the right cycle. However, such cases are rare, with circulatory death occurring within ${2}{\text { h}}$ in over ${70}{\%}$ of cases.^17^

The course of events following withdrawal of life-sustaining therapy (WLST), that all clinical protocols have to relate to, is illustrated in Fig. 2. The time between WLST and death is referred to as the agonal phase. The possibility of (short-term) survival,^17^ illustrated by the right cycle in Fig. 2, imposes legal and ethical restrictions on admissible treatments during, and leading up to, the agonal phase. Particularly, *ante-mortem* interventions should be motivated by the best interest of the patient and must not interfere with the possibility of (short-term) survival.

Measures for organ optimization mainly translate into reducing warm ischemic time. For heart organs, a distinction is made between warm ischemia in asystole and functional warm ischemia,^8^ where the greater metabolic needs of the latter make it more problematic in the DCD context.

### Hemodynamic Control in DCD

The onset of the agonal phase is typically associated with a cathecolamine “storm”, resulting in increased systemic resistance, and leading up to relative hypertension, possible tachycardia, and thus an increase in myocardial metabolism.^12^ Control of hemodynamic parameters is a potentially viable means of postponing the onset of IMC, and warm ischemic damage in general. Hemodynamic parameters available for pharmacological control include:

- vascular resistance (through arterial and venous tone);
- heart rate;
- myocardial contractility.

Cardiac output and tissue perfusion are directly dependent on the above three parameters. In this study we control these parameters with the goal of facilitating cardiac output after WLST, while limiting the associated cardiac work to avoid episodes of relative hypertension, tachycardia, and ischemia-induced ventricular fibrillation (VF). In this nominal work, we investigate whether the proposed methodology can serve to postpone the onset of ischemic myocardial contracture in a DCD large animal model.

Treatment associated with WLST needs to be delivered with dignity. To meet this need and the critical timing requirements imposed by the scenario, we have developed and demonstrated a fully automated feedback control system, in which a computer administers the delivery of intravenous drugs. The system adjusts the individual dosage of drugs in real-time based on the patient’s hemodynamic response, thus avoiding over- or under-dosing.

## Methods

### Cardiac Work Estimation

The hypothesis underlying the study is that the time between WLST and incidence of IMC is correlated with the ischemic work of the heart following WLST. To investigate this hypothesis, one under-estimating approximation

1 $${\underset{\raise0.3em\hbox{$\smash{\scriptscriptstyle-}$}}{W}} \propto \int _0^{T} P_{{\text{sys}}}(t)\;HR(t)\;{\text{d}}t,$$

and one over-estimating approximation

2 $$\overline{W} \propto \int _0^{T} P_{{\text{sys}}}^2(t)\;HR(t)\;{\text{d}}t,$$

of cardiac work were used where $t=0$ and $t=T$ denote the instances of WLST, and asystole or VF instance, respectively. $P_{sys}$ is the instantaneous systolic aortic pressure and *HR* is the instantaneous heart rate, defined at the instances of systolic peaks as $1/{\Delta t}$, where $\Delta t$ is the time passed since the preceding systolic peak. See Supplementary Sect. S1 for further details.

### Hemodynamic Control

We have developed and evaluated a feedback control system based on a computer-controlled infusion pump array; real-time invasive arterial pressure acquisition system; and a PC running software for measurement, control, actuation, logging, and associated graphical user interface. The base hardware has been described in previous works.^14^,^15^

The overall objective of the control system was to normalize vascular resistance between the instance of WLST and the incidence of circulatory collapse, defined in “Experimental Study” section, in order to facilitate cardiac output while limiting the amount of associated cardiac work. Individualized administration of the drugs is necessary to safely account for the variation in hemodynamic response between individuals after WLST. This motivates computer-controlled real-time adjustment of the timing and number of doses administered according to systolic aortic pressure measurements as opposed to using a fixed bolus protocol. From prior research,^14^ we have established the feasibility of normalizing systolic pressure using closed-loop computer control of noradrenaline and nitroglycerine. Nitroglycerine dosing was used as the control signal to decrease vascular resistance. Both bolus and continuous infusion dosing were considered in pilot experiments. It was concluded that bolus dosing was necessary to achieve sufficiently fast responses in $P_{sys}$. Based on pilot experiments described in Supplementary Sect. S4, the bolus size was set to ${1.5}{\text { mg}}$.

Pilot experiments indicated tachycardia and tolerance effects when exceeding three nitroglycerine boluses following WLST. If these were not sufficient to establish normotension, subsequent boluses of a synergistic calcium antagonist (nimodipine) and $\beta$-blocker (esmolol) mixture, comprising of nimodipine and esmolol, were administered. While counteracting both hypertension and tachycardia, the response time is slower, and the peak effect lower, compared to the nitroglycerine boluses.

Ventricular fibrillation was identified as the main contributor to IMC in our pilot experiments. To prevent this, a lidocaine bolus was given at the time of circulatory death, defined in “Experimental Study” section. A bolus dose of the calcium antagonist and $\beta$-blocker mixture were administered together with the lidocaine to prevent a prolonged episode of low-intensity myocardical work following circulatory death. The timing and doses of all drugs used in hemodynamic control are given in Supplementary Tab. S1 and S3.

A noradrenaline “safety” feedback controller was implemented for automatic drug infusion to counteract potential overdosing of nitroglycerine, otherwise resulting in hypotension. Systolic aortic pressure responses to constant noradrenaline infusions were recorded in three pilot experiments, and are shown in Supplementary Fig. S4.

Time-delayed first-order linear differential equation models were identified from the noradrenaline infusion responses shown in Supplementary Fig. S4, by minimizing the output error $\mathcal {L}_2$ norm. A proportional-integral-derivative (PID) controller was optimized for robust performance across these models. The optimization objective was to minimize the time from hypotension due to overdosing of nitroglycerine until acceptable systolic aortic blood pressure values were reached. A step disturbance was used to model the effect of nitroglycerine on the systolic aortic pressure. In the controller optimization, constraints were imposed to enforce robustness over the model set. More details of the controller design can be found in Supplementary Sect. S5.

To attenuate high-frequency measurement noise, a second-order low-pass filter was connected in series with the controller, resulting in the Laplace domain representation

3 $$\begin{aligned} K & = C F \\ C({\text{s}})&= k_{\text{p}}+k_{\text{i}}\frac{1}{s}+k_{\text{d}}s \\ F({\text{s}}) &= \frac{1}{(s T_{\text{f}} + 1)^2}\end{aligned}$$

where *C* is the PID controller, *F* is the low-pass filter and *s* is the Laplace variable. The PID parameters were $k_p = 9.63 \times 10^{-4}$ mg/h/mmHg, $k_i = 2.96 \times 10^{-5}$ mg/h/mmHg/s, $k_d = 8.14 \times 10^{-3}$ mg/h/mmHg s and $T_f = 2$ s. The controller was implemented with clamping anti-windup on the PC used in data acquisition and drug delivery actuation.

The set-point of this noradrenaline “safety” controller was set to ${70}{\text { mmHg}}$ for $P_{sys}$ during the first ${3}{\text { min}}$ following WLST, whereafter the controller was automatically deactivated.

### Experimental Study

The primary end-point was to study IMC occurrence ${60}{\text { min}}$ after circulatory death. The secondary end-point was the time between circulatory death and observed IMC.

Equal control and test group sizes of $n=6$ each were determined, based on ${70}{\%}$ anticipated ${60}{\text { min}}$ IMC incidence in the control group, and ${0}{\%}$ in the test group, at a false positive rate $\alpha = 0.05$, and false negative rate $\beta = 0.2$ (i.e., ${80}{\%}$ power). Inclusion criteria for both groups were defined to facilitate comparability of outcomes: stable hemodynamics at the time of WLST, with $P_{sys}\le$ 110 mmHg and $HR\le$ 110 min$^{-1}$; absence of agonal breathing following WLST; adherence to drug dosing protocol of the study; absence of anomalies at dissection. Details about animals excluded from the study and conducted pilot cases can be found in Supplementary Sect. S2.

Anesthesia was induced through intravenous injection of atropine, xylazin, and ketamine. Subsequently, midazolam and rocuronium were intravenously administered before placement of an endotracheal tube through tracheostomy. The animals were then mechanically ventilated using volume-controlled and pressure-regulated ventilation.

Upon introduction of intravenous propofol anesthesia, and intubation, the animals were instrumented with transducers to measure arterial and venous blood pressure and a 5-lead ECG. Arterial blood gas samples were collected and analyzed at baseline, and 1, 2,..., 5 min following WLST. The animals were given heparin to prevent coagulation. Doses and further details on the drugs are provided in Supplementary Sect. S3.

A neuromuscular blockade was established to prevent agonal breathing, whereupon WLST was performed. If the heart rate exceeded 110 bpm between WLST and circulatory collapse, a bolus of esmolol and nimodipine was given. Circulatory collapse was defined to occur at the first incidence of $P_{sys}<~{40}{\text { mmHg}}$. Circulatory death (cessation and irreversibility of cardiopulmonary function^19^) was defined as persistent circulatory collapse combined with arterial saturation remaining below s$_\text {a}$O$_2={30}{\%}$. Previous studies^2^,^5^ have associated a systolic pressure fall beneath ${50}{\text { mmHg}}$ with severe ischemia, motivating the choice of the lower systolic pressure limit. By this time, the animals was hypoxic with an arterial oxygen saturation well below ${30}{\%}$.^5^ Following a hands-off time of ${30}{\text { min}}$, sternotomy was performed, and the heart was inspected and palpated for IMC every ${5}{\text { min}}$ until ${60}{\text { min}}$ had passed since circulatory death. If there were palpable and visible signs of contracture in the left ventricular wall, IMC was confirmed. When IMC was verified or when ${60}{\text { min}}$ had passed since circulatory death, the heart was excised and transversely cut into slices as shown in Fig. 1. The heart was then dissected to inspect for anomalies that could have affected the outcome. To complement the qualitative diagnostic assessment with a quantitatively comparable measure, the left ventricular wall thickness was measured as the average thickness within a transverse plane halfway between the atrial-ventricular plane and the apex.

**Figure 3:**
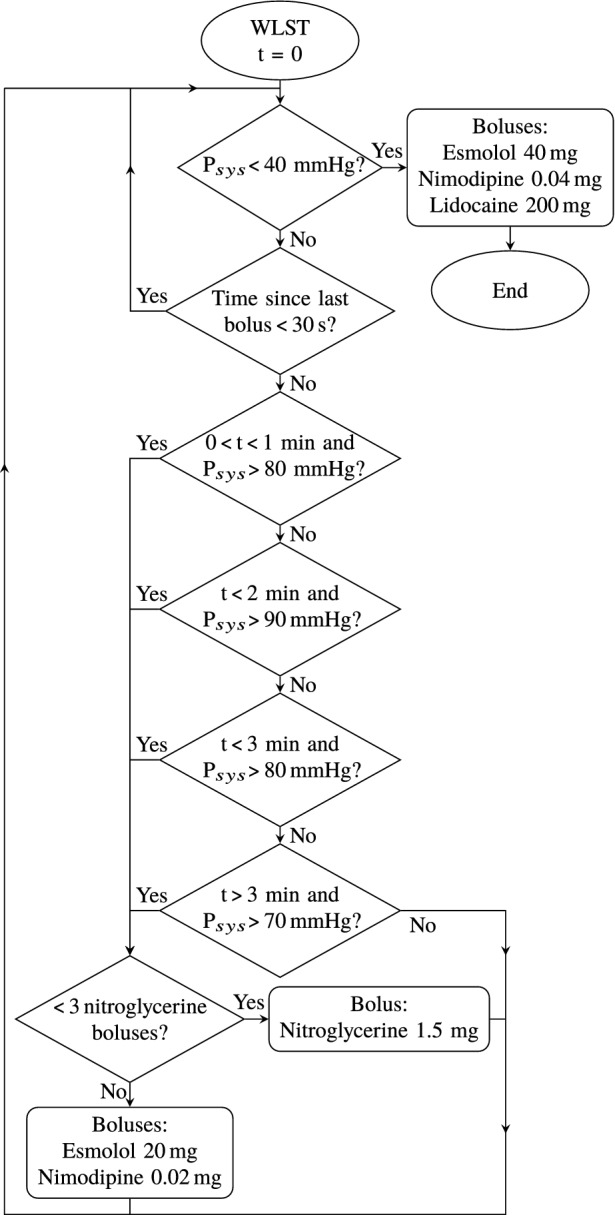
Flow chart illustrating the test group protocol. Time $t=0$ starts at the instance of WLST. The noradrenaline “safety” controller is activated during the first ${3}{\text { min}}$ following WLST, in order to avoid hypotension in case of low nitroglycerine tolerance. If the heart rate exceeded 110 bpm between WLST and circulatory collapse, an additional bolus of esmolol and nimodipine was given.

**Figure 4:**
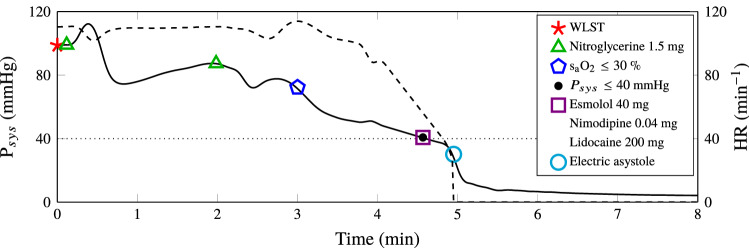
Representative test group experiment with fully automated drug dosing according to the protocol illustrated in Fig. 3. Systolic pressure, $P_{sys}$ is shown solid and heart rate, *HR*, in dashed. Markers indicate events according to the figure legend. The dotted black line indicates the systolic pressure associated with circulatory collapse.

The test and control group protocols were identical with the exception of the test group being subjected to the hemodynamic control protocol described in “Hemodynamic Control” section. The test group protocol is illustrated through the flowchart in Fig. 3. Drugs used in the test group protocol were nitroglycerine, noradrenaline, lidocaine, esmolol and nimodipine, see Fig. 3 and Supplementary Tab. S1 and S3 for details about bolus dosing and timing. Noradrenaline was administered by the aforementioned “safety” feedback control system using an Alaris TIVA infusion pump. Bolus doses of the other drugs were manually administered, due to a lack of remote-controlled bolus capability of the Alaris TIVA pumps. This was later implemented, and two additional fully automated cases, one illustrated in Fig. 4, were successfully completed.

## Results

The investigated method for normalization of hemodynamics upon WLST with the aim to facilitate DCD procurement of hearts resulted in none of the six test group individuals developing IMC within ${60}{\text { min}}$ of warm ischemia following circulatory death. All six control group individuals developed IMC within ${60}{\text { min}}$, with four having developed IMC by the time of sternotomy, ${30}{\text { min}}$ following circulatory death.

Figure 1 shows representative cross sections of two hearts from the study: (a) was procured from a test group animal ${60}{\text { min}}$ following circulatory death; (b) from a control group animal ${30}{\text { min}}$ following circulatory death. The heart in (a) shows no signs of IMC, while IMC is fully developed in (b), as seen by the severely restricted left-ventricular lumen. The average left ventricular wall thickness, measured half-way between the atrial-ventricular plane and the apex at the time of dissection, was 10 mm (range 8–16) within the test group and 20 mm (range 16-22) within the control group.

**Figure 5:**
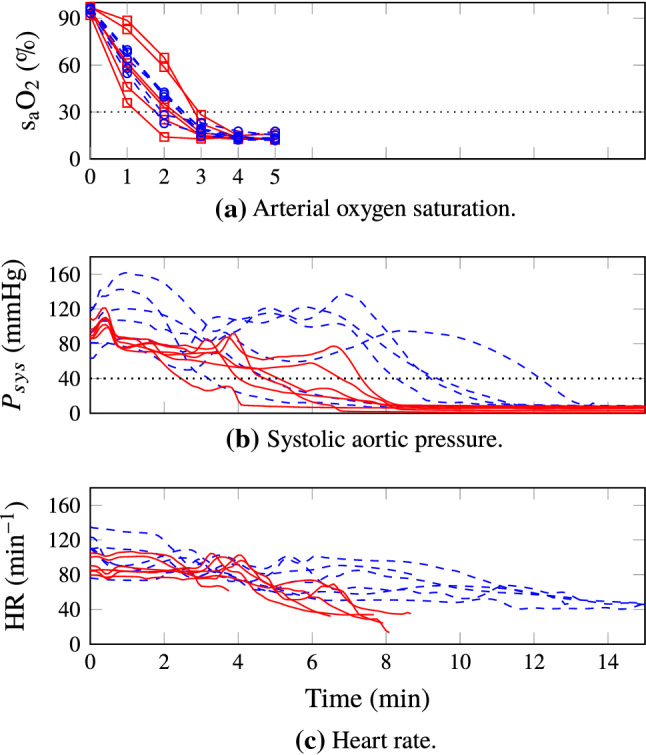
Test (solid red, squares) and control (dashed blue, circles) group hemodynamic responses following withdrawal of life-sustaining therapy (WLST) at time ${0}{\text { min}}$. The dotted horizontal line in (a) indicates oxygen saturation s$_\text {a}$O$_2=$ 30%. The dotted horizontal line in (b) indicates the systolic aortic pressure, $P_{sys}={40}{\text { mmHg}}$, associated with circulatory collapse. Circulatory death was defined to occur when both s$_\text {a}$O$_2<{30}{\%}$ and $P_{sys}<{40}{\text { mmHg}}$ were fulfilled.

**Figure 6:**
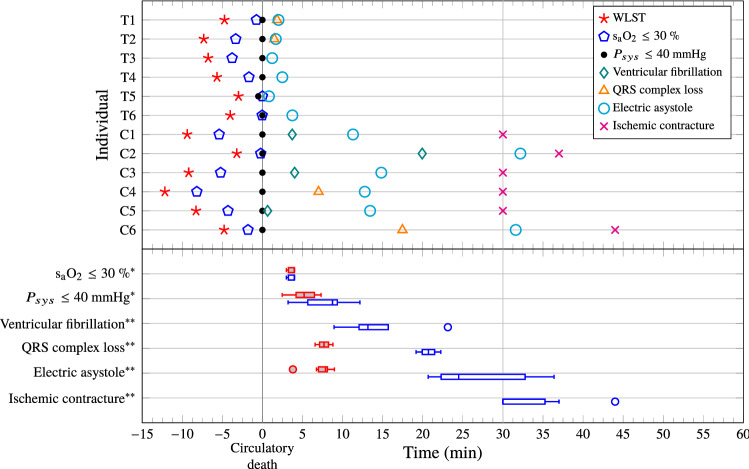
Distribution of events. The top part shows events for test (T) and control (C) group individuals. QRS complex loss markers have been omitted for individuals where QRS complex loss coincided with ventricular fibrillation or electric asystole. IMC incidence has been reported as 30 min if IMC was observed at the instance of sternotomy, which was performed 30 min after circulatory death. The bottom part shows the distribution of events within the study groups. Filled red boxes are used for the test group; empty blue ones for the control group. In absence of incidence, IMC and ventricular fibrillation statistics are not presented for the test group. Note that the distributions of arterial saturation s$_\text {a}$O$_2<30$ %, and systolic pressure $P_{sys}<40$ mmHg, are reported with the withdrawal of life-sustaining therapy (WLST) time instance as zero reference, while all other events are reported with the time instance of circulatory death as zero reference.

Figure 5 shows the hemodynamic responses for all test and control subjects, following withdrawal of life-sustaining therapy at $t=0$ min. The markers in Fig. 5a show oxygen saturation (s$_\text {a}$O$_2$) of arterial blood gas samples. The dotted horizontal line corresponds to s$_\text {a}$O$_2={30}{\%}$. All individuals reached an arterial saturation below ${30}{\%}$ within ${3}{\text { min}}$ following WLST. The mean ± standard deviation durations between WLST and occurrence of s$_\text {a}$O$_2={30}{\%}$, linearly interpolated between samples, were $133\pm {38}{\text {s}}$ in the test group and $143\pm {27}{\text {s}}$ in the control group. Mean ± standard deviation desaturation rates were $-29 \pm {4}{\%/\text {min}}$ in the test group and $-33 \pm {12}{\%/\text {min}}$ in the control group. This indicates similar metabolic rates between the groups. Systolic aortic pressures ($P_{sys}$) are shown in Fig. 5b. The dotted horizontal line corresponds to $P_{sys}={40}{\text { mmHg}}$, indicating the systolic pressure associated with circulatory collapse. Heart rates (HR), computed from ECG RR-intervals until loss of QRS-complex, or onset of VF, are shown in Fig. 5c. Per-individual events are shown in the top part of Fig. 6, in which time zero corresponds to circulatory death. The bottom part visualizes the temporal distribution of events.

There was no notable overdosing of nitroglycerine in any of the cases. Consequently, the noradrenaline controller administered only very small drug doses in two cases: 1.1 $\mu$g, beginning ${149}{\text { s}}$ in T4 after WLST; 6.5 $\mu$g in T6, beginning ${113}{\text { s}}$ after WLST.

**Figure 7:**
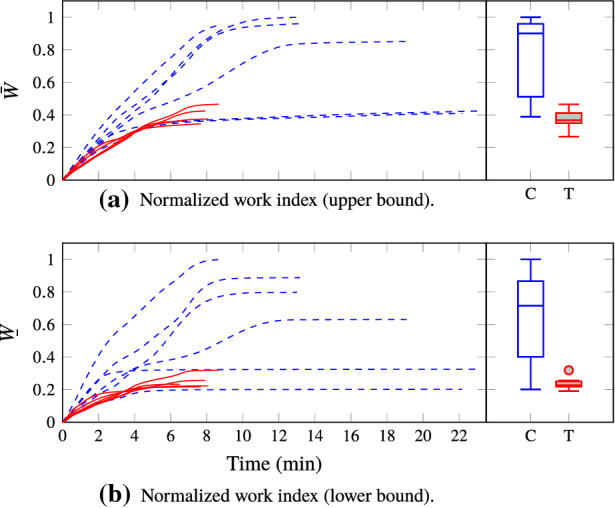
Normalized work indices between withdrawal of life-sustaining therapy (WLST) at time 0 min and the incidence of either asystole or ventricular fibrillation are shown to the left. Test group estimates are shown in solid red; control group estimates in dashed blue. The two lowermost curves in the control group correspond to individuals C2 and C6 in Fig. 6, which showed circulatory collapse less than five minutes after WLST. Data computed using (1) is shown in (a) and using (2) is shown in (b). The normalized work index final value distributions of the control group (C, empty blue boxes) and test group (T, filled red boxes) are shown to the right.

Work indices ${\underline{W}}$ and $\bar{W}$ for all individuals are shown in Fig. 7. The distributions of their final values are shown to the right in the same figure. The median decrease in work indices between control and test group was ${59}{\%}$ for ${\underline{W}}$ and ${68}{\%}$ for $\bar{W}$.

The single-sided Mann-Whitney test reveals a significant ($p<0.002$) difference in IMC incidence ${60}{\text { min}}$ after withdrawal of life-sustaining therapy between the groups.

## Discussion

This study demonstrated that it is feasible to postpone ischemic myocardial contraction up to ${1}{\text { h}}$ following circulatory death, through automatic control of hemodynamic drug delivery.

However, further investigation needs to be undertaken to see if the asystolic non-contracted heart may be reconditioned to partial or full function after ${30}{\text { min}}$ or ${60}{\text { min}}$ of circulatory arrest *in situ* at normothermia. Histological comparison, *ex vivo* functional evaluation, and ultimately transplantation are markers suggested for further evaluation to determine feasibility for transplantation. A method to recondition and preserve porcine hearts has been developed in-house.^13^,^16^ Safe orthotopic transplantation was done with hearts extracted ${24}{\text { h}}$ after brain death and kept vital by non-ischemic-heart-perfusion (NIHP) for ${24}{\text { h}}$.^16^ To validate non-contracted hearts up to ${1}{\text { h}}$ after circulatory death, we plan to do NIHP, followed by orthotopic transplantation. If the function of such hearts is good, this method may support broader clinical implementation of heart transplantation following withdrawal of life-sustaining therapy (controlled DCD).

While it is possible to implement the protocol manually in a controlled lab environment with well-rehersed personnel, its clinical feasibility is low, taking into account the narrow timing requirements and the requirement of calm and dignity in the presence of next of kin. The use of a feedback control system solves both these problems. Clinical implementation relies only on standard ICU monitoring and intravenous access, both of which can be expected in the considered patient category.

The controlled DCD model used in this work is similar to the clinical scenario.^20^ However, unlike the clinical scenario, the animals had not suffered neurological damage. Measures were therefore taken to establish a standardization of the agonal phase, further explained in Supplementary Sect. S3, manifested in the small variability in desaturation profiles shown in figure Fig. 5a. While the test group protocol was designed to include only drugs broadly accepted in the considered context, local protocols can affect admissibility of certain drugs. We have found no reason to believe that the test group results could not have been obtained by means of another set of drugs with similar hemodynamic effects, as long as timing and dosing are appropriately chosen.

There is no internationally recognized definition of circulatory death. It is therefore debatable whether the definition used in this work (s$_\text {a}$O$_2 <{30}{\%}$ and $P_{sys}<{40}{\text { mmHg}}$) would gain broad acceptance. However, the conclusion of the study would be the same with slightly differing definition of circulatory death (based on the same parameters), as can be verified by studying the profiles of Fig. 5.

As seen in Fig. 5, and reported in other preclinical studies,^5^,^11^ the time between WLST and circulatory collapse is generally shorter than in clinic, where it is usually around 15–20 min, in absence of agonal breathing.^9^ Furthermore, the circulatory collapse process progressed somewhat more rapidly in the test group. This was observed during the study, once the test and control group protocols had been fixed. In subsequent experiments we made the automated drug delivery less aggressive, and increased the systolic pressure setpoint for the noradrenaline “safety” controller during the initial phase following WLST. With these changes to the test group protocol, we were able to postpone IMC beyond 1 h with hemodynamic trajectories like those of the study controls.

The final values of the work indices in Fig. 7 and corresponding IMC onset times in Fig. 6 indicate that the work indices constitute useful predictors of IMC onset. Circulatory collapse occurred after less than five minutes in two of the control group individuals: C2 and C6 in Fig. 6. This resulted in work indices similar to those representative for the test group and a later occurrence of stone heart (${37}{\text { min}}$ and ${44}{\text { min}}$). Relatedly, the distinct difference in final work indices between the control and test group shown in Fig. 7 indicates that the investigated protocol for pharmacological normalization of hemodynamics is effective in postponing the onset of IMC following WLST in the considered large animal model.

## Conclusion

A pharmacological method, intended to postpone the onset of ischemic myocardial contracture (IMC), with the aim to facilitate controlled DCD procurement of hearts, was developed and evaluated. None of the six test group animals developed IMC within ${60}{\text { min}}$ of warm ischemia, following circulatory death caused by withdrawal of life-sustaining therapy. All six control group animals developed ischemic myocardial contracture within ${60}{\text { min}}$ following circulatory death, with four having developed IMC by the time of sternotomy, ${30}{\text { min}}$ following circulatory death. This demonstrates pre-clinical feasibility of the proposed method, and motivates further research aimed at adapting it for the clinical setting. Further studies are needed to investigate whether the function of the heart can be fully restored.

## Supplementary Information

Below is the link to the electronic supplementary material.

Electronic supplementary material 1 (PDF 150 kb)
