# Supplementary material for: Prevention of Ischemic Myocardial Contracture Through Hemodynamically Controlled DCD
Source: Cardiovasc Eng Technol. 2021 Apr 29;12(5):485–93. doi: 10.1007/s13239-021-00537-8 (PMC8481220; doi:10.1007/s13239-021-00537-8)
Supplement: Supplementary file 1 — Electronic supplementary material 1 (PDF 150 kb) [file 13239_2021_537_MOESM1_ESM.pdf]

Supplement

Prevention of ischemic myocardial contracture through hemodynamically controlled DCD

Cardiovascular Engineering and Technology

Ylva Wahlquist[1], Kristian Soltesz[1], Qiuming Liao[2], Xiaofei Liu[3], Henry Pigot[1], Trygve Sjöberg[2] and Stig Steen[2]

[1] Wahlquist, Soltesz and Pigot are with the Department of Automatic Control, Lund University, Lund, Sweden. Correspondence to Ylva Wahlquist, E-mail: ylva.wahlquist@control.lth.se

[2] Liao, Sjöberg and Steen are with the Division of Thoracic Surgery, Department of Clinical Sciences, Lund University, Sweden and the Department of Cardiothoracic Surgery, Skåne University Hospital, Sweden

[3] Liu is with the First Affiliated Hospital of Zhengzhou University, Zhengzhou, China

S1 Cardiac work estimation

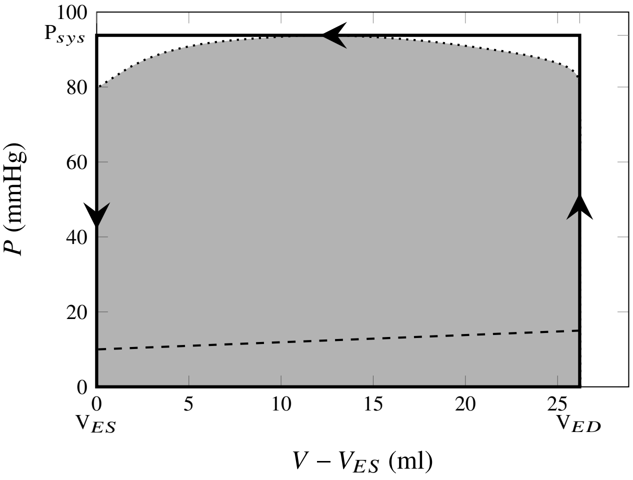

Fig. S1 Systolic pressure-volume (PV) path (dotted) between end-diastole, (ED, mitral valve closure), and end-systole, (ES, aortic valve closure), was recorded in a 30 kg pig during one pilot experiment, represented Fig. S2. The left-ventricular diastolic PV path, from ES to ED, of the cardiac cycle (dashed) is only qualitatively illustrated, and not used when considering the entire heart, rather than the isolated left ventricle. The net work performed by the left-ventricle during the cardiac cycle is the area between the dotted and dashed lines; corresponding net work performed by the heart is quantified by the grey area. The area enclosed by the solid line is the proposed cardiac cycle work estimate, $\hat{W}_c$, defined through (S4).

Left ventricular pressure-volume (PV) loops are frequently used in clinical and research cardiology. The PV loop shows ventricular pressure plotted against ventricular volume over one or several cardiac cycles, providing a contour similar to the one enclosing the grey area of Fig. S1. Integrating pressure over volume in the PV loop over one cardiac cycle yields the work exerted *on* the system. The work exerted *by* the system is hence

$$W(V_1, V_2) = -\int_{V_1}^{V_2} P(V)\, dV, \tag{S1}$$

when transitioning from a volume V_1 to another volume V_2 along the PV contour. To be accurate, (S1) provides an upper bound for this work, and that bound is tight for lossless systems. This assumption is tacitly made in cardiac PV loop analysis, where the area enclosed by the PV contour is defined to be the stroke work. It is, however, not valid during ventricular fibrillation (VF), in which considerable energy is consumed without resulting in net blood transport, and hence no work is done according to (S1).

Letting t_1 be the time at which the volume is V_1, i.e., $V(t_1) = V_1$, and similarly defining t_2 through $V(t_2) = V_2$, the work of (S1) can be expressed as

$$W(t_1, t_2) = -\int_{t_1}^{t_2} P(t)\frac{dV}{dt} dt = -\int_{t_1}^{t_2} \varphi(t)P(t)\, dt, \tag{S2}$$

where $\varphi(t) = \dot{V}(t)$ is the volumetric flow rate entering the left ventricle. When $W > 0$, the left ventricle exerts work *on* the blood; when $W < 0$, the blood exerts work on the left ventricle. Since $P > 0$ throughout the cardiac cycle, $W > 0$ holds whenever left ventricular volume decreases ($\dot{V}(t) < 0$, characterized by the dotted line in Fig. S1), and work is exerted by the blood whenever left ventricular volume increases: $\dot{V}(t) > 0$, characterized by the dashed line in Fig. S1. The relatively small fraction of work exerted *by* the blood, quantified by the area under the dashed line in Fig. S1, can be viewed as a free contribution when the left ventricle is considered as an isolated system, which is commonly the case in PV loop analysis. However, when considering the entire heart, this contribution is not free: it is the combined contribution of the atria and the right ventricle.

Under the valid assumption that central venous pressure is low compared to aortic pressure, the work W_c, exerted by the heart during one cardiac cycle can thus be expressed

$$W_c = \int_{t_{ED}}^{t^{ES}} P(t)\, CO(t)\, dt, \tag{S3}$$

where P is the (instantaneous) aortic pressure, t_{ED} the end-diastolic time instance, t_{ES} the end-systolic time instance, and $CO(t) = -\varphi$ the (instantaneous) cardiac output.

A fair approximation of W_c, $\hat{W}_c$, is obtained by replacing the contour segment between V_{ED} and V_{ES} with the systolic isobar in Fig. S1, P_{sys}. Then (S3) is approximated by

$$\hat{W}_c = P_{\text{sys}}\, SV, \tag{S4}$$

where $SV = V_{ES} - V_{ED}$ is the stroke volume. The estimate $\hat{W}_c$ corresponds to the area enclosed by the solid line in Fig. S1. The total energy consumption between WLST and the incidence of either asystole or VF is the sum of the individual W_c contributions.

A reasonable assumption is that SV is positively correlated with P_{sys}. This will be expressed as $SV(P_{sys})$, where $SV : \mathbb{R}^{+} \mapsto \mathbb{R}^{+}$ is some bounded monotonously non-decreasing function. The resulting relative difference in estimated work (S4) between cardiac cycles with systolic pressures P_a and $P_b < P_a$ is

$$\frac{P_a SV(P_a) - P_b SV(P_b)}{P_a SV(P_a)} = 1 - \frac{P_b}{P_a}\frac{SV(P_b)}{SV(P_a)}. \tag{S5}$$

The properties of $SV(P_{sys})$, combined with $P_b < P_a$, yield $SV(P_b) \leq SV(P_a)$, with equality when $SV(P_{sys}) \propto 1$, i.e., when SV is not increasing with P_{sys}.

To justify this assumption, the nature of $SV(P_{sys})$ was investigated through two pilot experiments, where a transit-time flow meter, connected to the LabChart system detailed in Sect. S3, was secured across the aorta upon sternotomy, and otherwise adhering to the control group protocol. This enabled computing SV as the time integral of flow over each cardiac cycle. The outcome is shown in Fig. S2, confirming that the monotonicity assumption on $SV(P_{sys})$ is sound. Furthermore, it suggests that while the conservative estimate $\underline{W}$ is accurate for the saturated region (almost horizontal segment of blue triangles), the non-saturated region (the remainder of plotted data) is better approximated by $SV(P_{sys}) \propto P_{sys}$.

A lower bound of the estimate is therefore obtained by assuming that SV is independent of P_{sys}, resulting in the total work estimate

$$\underline{W} \propto \int_0^T P_{sys}(t)\, HR(t)\, dt, \tag{S6}$$

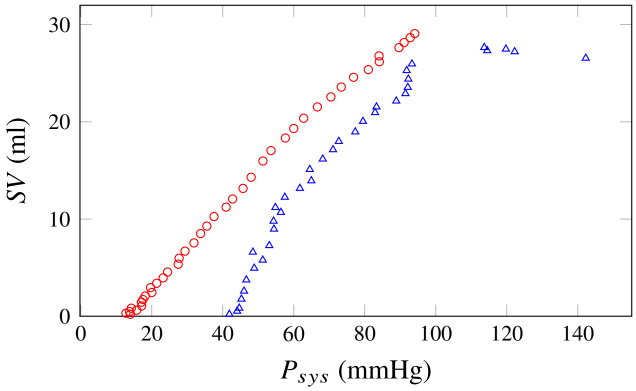

Fig. S2 Relationship between systolic aortic pressure, P_{sys}, and stroke volume, SV, measured in 30 kg animals during two pilot experiments: one illustrated by red circles; the other by blue triangles.

where $t = 0$ and $t = T$ denote the instances of WLST, and asystole or VF instance, respectively. HR is the instantaneous heart rate, defined at the instances of systolic peaks as $1/\Delta t$, where Δt is the time passed since the preceding systolic peak.

The pilot experiments also suggest an upper bound on the total work estimate

$$\bar{W} \propto \int_0^T P_{sys}^2(t)\, HR(t)\, dt, \tag{S7}$$

based on SV being proportional to P_{sys}.

Since $\underline{W}$ and $\bar{W}$ are defined only up to proportionally, they will be denoted work *indices* rather than *estimates*, and their utility is limited to comparison between individuals or groups, within which cardiovascular physiology is similar. The central hypothesis, investigated in the study, is that the time integrals of these indices following WLST provides useful predictions of IMC incidence, and thus suggest how pharmacological normalization of hemodynamics can serve to prevent IMC.

Evaluation of the estimation of the work over one cardiac cycle (S4) of the main manuscript relies on SV, or equivalently CO, being measured. However, pilot experiments revealed that manipulation of the ischemic heart easily triggers the onset of IMC. Placement of a PV loop catheter was consequently ruled out as an option for the controlled study. Similarly, the pleural access required to place an ultra-sonic flow probe over the aorta was found to result in (partial) collapse of the lungs, suspected to affect the desaturation process. Consequently, WLST was performed with intact thorax during the study.

S2 Excluded animals and pilot cases

Three animals were excluded, all from the test group, for the following reasons: insufficient curarization resulting in agonal breathing; systolic pressure rise prior to WLST (the experiment was concluded and IMC was absent 60 min after circulatory death); nitroglycerine administration exceeding protocol limit.

In addition to the 12 included plus 3 excluded study cases, 11 pilot cases were conducted: 6 to obtain modeling data for controller design, 3 to determine adequate doses of the drugs that were not computer-controlled, and 2 to investigate the $SV(P_{sys})$ relationship explained in Supplementary Sect. S1. Pilot experiment and excluded animals fell within the same weight range as those included in the study.

S3 Study details

The animals were mechanically ventilated using volume-controlled (4 l minute volume at 18 breaths/min) and pressure-regulated ventilation (5 cmH_2O PEEP). Inspired oxygen fraction was 21 %, and end tidal CO_2 was kept between 4.5 kPa and 5.5 kPa. Details of drugs and equipment used in the experiments can be found in Table S1 and S2.

Three venous catheters were secured into the superior vena cava with their tips at the level of the right atrium. These catheters were used for anesthesia maintenance, continuous venous pressure monitoring and manual drug administration. Two catheters of the same type were secured into the ascending aorta for online arterial pressure monitoring and blood gas sampling. Arterial blood gas samples were collected and analyzed at baseline, and $1, 2, \ldots, 5$ min following WLST.

Blood pressure transducers were connected to the arterial and venous catheters, and intermittently flushed with saline solution. Separate transducer pairs were used for the feedback control system and visualization. Both systems logged to file, results presented herein are from the LabChart logs. A 5-lead ECG was also connected to LabChart, running on a Windows 7 PC.

Rocoronium was infused at 60 mg/h, with an additional 50 mg bolus just before WLST, to prevent agonal breathing. Without complete neuromuscular blockade, one would expect a much larger spread between desaturation profiles, prompting increased study group sizes in order to draw valid comparative conclusions. Rocuronium was therefore administered to establish a neuromuscular blockade, and the endotracheal tube was clamped at the instance of WLST. Both these measures should be viewed as parts of the large-animal model, and not the proposed DCD protocol. They guarantee a total absence of gas exchange.

Bolus dose timing for the test group individuals are shown in Table S3.

Table S1: Complete list of drugs used in the main study, together with corresponding dose and manufacturer specifications. Saline solution was administered to compensate for dehydration losses.

Drug	Dose	Full drug name and manufacturer
atropine	0.5 mg	Atropin, Mylan AB, Stockholm, Sweden
esmolol	20 mg/bolus	Brevibloc, Baxter Medical AB, Kista, Sweden
heparin	25000 IU	Heparin, LEO Pharma AB, Malmö, Sweden
ketamine	750 mg	Ketaminol vet, Intervet, Boxmeer, Netherlands
lidocaine	200 mg	Xylocard, Aspen Nordic, Dublin, Ireland
midazolam	25 mg	Midazolam, Panpharma S.A, Trittau, Germany
nimodipine	0.02 mg/bolus	Nimotop, Bayer Healthcare AG, Leverkusen, Germany
nitroglycerine	1.5 mg/bolus	Nitroglycerin, BioPhausia AB, Stockholm, Sweden
noradrenaline	computer controlled	Noradrenalin, Pfizer AB, Sollentuna, Sweden
propofol	4 mg/kg/h	Propofol-Lipuro, B. Braun Medical AB, Melsungen, Germany
rocuronium	20 mg, 60 mg/h	Rocuronium, Fresenius Kabi, Graz, Austria
xylazin	100 mg	Rompun vet, Bayer AB, Solna, Sweden

Table S2: Instrumentation for measurement and drug delivery

Equipment	Name	Manufacturer
Flow meter	Flowmeter CM4000	CardioMed, Lindsay, Canada
Ventilator	Servo Ventilator 300	Siemens AB, Solna, Sweden
Venous catheter	Secalon-T	Merit Medical, Singapore
Blood gas analyzer	ABL 700	Radiometer, Copenhagen, Denmark
Blood pressure transducer	Meritrans DTXPlus	Merit Medical, Singapore
DAQ	PowerLab 16/35	AD Instruments, Colorado Springs, CO
Infusion pump	Alaris TIVA	BD, Franklin Lakes, NJ
LabChart 8	AD Instruments	Colorado Springs, CO

Table S3: Bolus dose timing for test group individuals T1–T6, in seconds measured from WLST.

	Nitroglycerine			Esmolol and Nimodipine			Lidocaine, Esmolol and Nimodipine (P_{sys} = 40 mmHg)
	#1	#2	#3	#1	#2	#3	#1
T1	5	83	212	233			292
T2	3	81	194	216	260	292	296
T3	6			347			409
T4	177			207			350
T5	11	73					165
T6	12	126	169	192			233

S4 Nitroglycerine bolus dose

Aortic systolic pressure responses to nitroglycerine, collected during two pilot experiments, are shown in Fig. S3. The experiments indicated that a bolus dose of 1 − 2 mg results in a sufficient response, and that there is an apparent saturation in the vasodilative effect beyond this dose. Dose size was therefore fixed to 1.5 mg throughout the main study.

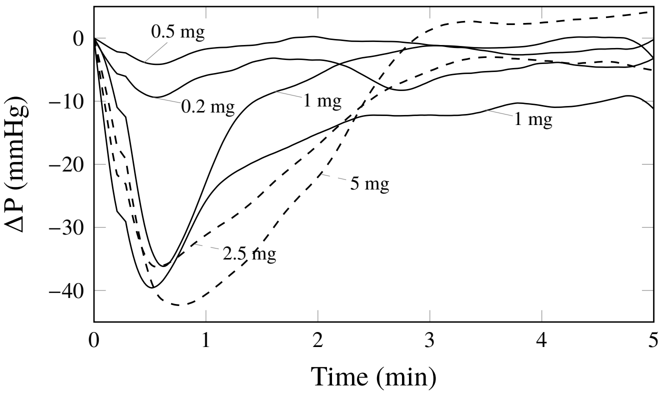

Fig. S3 Systolic pressure deviations, $\Delta P_{sys} = P_{sys} - P_0$, resulting from a nitroglycerine bolus at time zero, where P_0 denotes the baseline value of the systolic pressure P_{sys}. The responses were obtained through two pilot experiments on one 30 kg pig (dashed) and one 35 kg pig (solid).

S5 Noradrenaline controller

A noradrenaline "safety" controller was implemented for automatic drug infusion to counteract potential overdosing of nitroglycerine, otherwise resulting in hypotension. Systolic aortic pressure responses to constant noradrenaline infusions were recorded in three pilot experiments, and are shown in Fig. S4. These step responses for noradrenaline were used for identification of low-order models for the dynamics. A first-order model with time delay

$$P(s) = \frac{b}{s+a}e^{-sL} \tag{S8}$$

is sufficient to describe the drug response dynamics. The model parameters a, b, L were identified by minimizing the output error $\mathcal{L}_2$ norm, with the error being the difference signal between measured response and corresponding model output. The resulting model parameters can be found in Table S4. A discrete time version (zero-order hold with 1 s sampling period) of the filtered PID controller was then synthesized based on a modification of the methodology in [22]. A second-order filter F was chosen to guarantee high frequency roll-off, and the filter time constant was fixed to $T_f = 2$ s, upon inspection of measurement noise characteristics in the systolic pressure signal.

The PID parameters kp, k_i, k_d of (3) were optimized by minimizing settling time following a step output disturbance, modeling a sudden change in systolic pressure. The minimization was performed such that the maximum closed-loop 2 % settling time across the identified models was minimized. The minimization was subject to a constraint, limiting the response overshoot to 50 % of the amplitude of the disturbance step amplitude, across all identified models. This constraint was introduced to limit hypertension peaks and introduce additional robustness to the closed-loop system. The resulting optimized PID parameters were $k_p = 9.63 \cdot 10^{-4}$ mg/h/mmHg, $k_i = 2.96 \cdot 10^{-5}$ mg/h/mmHg/s, and $k_d = 8.14 \cdot 10^{-3}$ mg/h/mmHg·s.

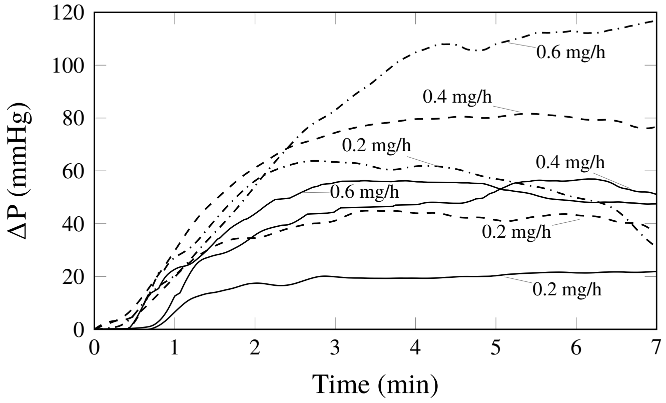

Fig. S4 Systolic pressure deviations, $\Delta P_{sys} = P_{sys} - P_0$, resulting from a noradrenaline infusion at time zero, where P_0 denotes the baseline value of the systolic pressure P_{sys}. The responses were obtained through pilot experiments using three pigs, two weighing 30 kg (solid and dashed) and one weighing 35 kg (dot-dashed)

Table S4: First order model parameters with time delay (S8), identified from noradrenaline infusion responses in Fig. S4

a	b	L
0.013	1.49	22.00
0.014	1.92	42.25
0.023	1.94	33.11
0.020	4.28	26.99
0.015	3.04	26.60
0.061	12.47	34.87
0.014	2.48	54.68
